# Real‐World Outcomes of Nivolumab and Ipilimumab in Metastatic Melanoma as Third Line and Beyond

**DOI:** 10.1002/ijc.70520

**Published:** 2026-04-28

**Authors:** Yago Garitaonaindia, Søren Kjær Petersen, Louise M. Guldbrandt, Troels H. Borch, Christina H. Ruhlmann, Rasmus Blechingberg Friis, Adam A. Luczak, Lars Bastholt, Henrik Schmidt, Inge Marie Svane, Eva Ellebaek, Marco Donia

**Affiliations:** ^1^ National Center for Cancer Immune Therapy (CCIT‐DK), Department of Oncology, Copenhagen University Hospital Herlev Denmark; ^2^ Department of Oncology Odense University Hospital Odense Denmark; ^3^ Department of Oncology Aarhus University Hospital Aarhus Denmark; ^4^ Department of Oncology Aalborg University Hospital Aalborg Denmark

**Keywords:** immunotherapy, melanoma, nivolumab/ipilimumab, real world data

## Abstract

Nivolumab plus ipilimumab has demonstrated activity after anti‐PD‐1 failure in advanced melanoma, but its effectiveness in later lines and as rechallenge remains unclear. We aimed to characterize outcomes of nivolumab/ipilimumab administered in the third line or beyond. Using the Danish Metastatic Melanoma Database (DAMMED), we identified patients with metastatic melanoma (excluding uveal melanoma) treated with nivolumab/ipilimumab after at least two prior lines of therapy, including adjuvant treatment, between 2017 and 2024. Baseline characteristics, prior treatments, and clinical outcomes were collected. Seventy‐three patients were included (median age 57.8 years), of whom 47.9% had brain metastases. Most had progressed on anti‐PD‐1‐based therapy (93.2%); 32.9% had prior exposure to anti‐CTLA‐4, and 84.9% had received BRAF/MEK inhibitors. Nivolumab/ipilimumab was administered as third‐line therapy in 71.2%. After a median follow‐up of 27.6 months, the overall response rate was 23.3% (12.5% with prior anti‐CTLA‐4 exposure vs. 28.6% without). Median duration of response was 19.4 months (95% CI, 14.5‐NR). Median PFS was 2.7 months (95% CI, 2.4–5.7) and median OS was 9.6 months (95% CI, 6.5–20.1). In conclusion, in heavily pretreated melanoma, nivolumab/ipilimumab induces durable responses in a minority of patients, with reduced efficacy after prior anti‐CTLA‐4 exposure.

## Abstract (highlights)

What's new?

Progression after anti‐PD‐1‐based therapy is a predominant scenario in metastatic melanoma. However, real‐world data informing treatment of metastatic melanoma in later lines remains scarce. This retrospective nationwide, population‐based cohort study shows in a real‐world setting that nivolumab plus ipilimumab can induce durable responses beyond the second line in a small but clinically meaningful subset of heavily pretreated patients, even with brain metastases. Prior exposure to both anti‐PD‐1 and anti‐CTLA‐4 markedly reduced response likelihood, and prior benefit did not predict re‐induction outcomes. The findings fill a critical evidence gap and define realistic expectations for late‐line immunotherapy.

## Graphical abstract

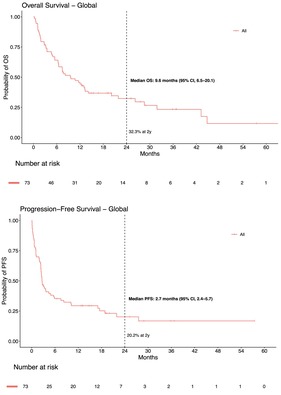

**Abbreviations**
BOR: best objective response
CR: complete response
DCR: disease control rate
DoR: duration of response
HR: hazard ratios
IQR: interquartile range
MM: metastatic melanoma
ORR: overall response rate
OS: overall survival
PFS: progression‐free survival
PR: partial response
SD: stable disease
TIL: tumor‐infiltrating lymphocytes

## 1 Introduction

Patients with progression on or after anti‐PD‐1 based therapy represent today a predominant scenario in metastatic melanoma (MM), yet randomized data to inform post‐PD‐1 management are scarce. Tumor‐infiltrating lymphocyte (TIL) therapy has demonstrated clinical activity in patients resistant to anti‐PD‐1 therapy (86% of patients in the pivotal trial cohort had progressed on PD‐1), achieving meaningful ORR and PFS, although feasibility and toxicity concerns remain [1]. A phase II trial further showed that nivolumab/ipilimumab had superior PFS and ORR compared to ipilimumab monotherapy in patients with immediate progression after anti‐PD‐1 therapy [2]. These findings, consistent with a single‐arm prospective study and a retrospective analysis [3, 4], have positioned nivolumab/ipilimumab as the most widely adopted treatment option after progression on anti‐PD‐1 monotherapy. Evidence after treatment with combined or sequential anti‐PD‐1 plus anti‐CTLA‐4, except BRAF/MEK inhibitors for patients with tumors harboring a *BRAF* mutation, is very scarce. Subsequent lines of treatment are not evidence based and may include clinical trials or rechallenge either with immunotherapy or BRAF/MEK inhibitors [5]. Evidence is also lacking regarding the role of nivolumab/ipilimumab in such a heavily pretreated patient population, including its potential use as a rechallenge after prior exposure.

The present study aims to evaluate the activity of nivolumab/ipilimumab in patients with MM treated in the third or later line within a real‐world setting.

## 2 Methods

### 2.1 Patient Selection and Data Acquisition

We conducted a retrospective, population‐based cohort study using the DAMMED, a nationwide population‐based registry with prospective data collection estimated to capture more than 95% of all MM cases in Denmark [6]. Patients with unresectable stage III or stage IV melanoma, excluding those with uveal melanoma, and who initiated nivolumab/ipilimumab after at least two prior lines were considered. For this purpose, adjuvant/neo‐adjuvant treatment with anti‐PD‐1 was considered a treatment line. The inclusion period was between January 2017 and September 2024. Patients were followed until September 6th, 2025 (data cut‐off), death, or last contact, whichever came first, with a minimum follow‐up of 12 months.

We collected data regarding baseline patient and tumor characteristics, previous (neo)adjuvant treatment, clinical outcomes, and information on other treatments for MM.

All patients were staged according to AJCC classification 8th edition [7]. Objective responses in DAMMED are assessed by the investigators according to RECIST 1.1, based on standard radiology reports. Additional quality control was conducted by the authors S.K.P., R.B.F., A.A.L., L.B., E.E., and M.D.

### 2.2 Outcome Measures

Progression‐free survival (PFS) was defined as the time from treatment initiation until progression or death, and overall survival (OS) as the time until death from any cause. Overall response rate (ORR) was defined as the proportion of patients achieving the best objective response (BOR), either complete response (CR) or partial response (PR), and disease control rate (DCR) as CR, PR, or stable disease (SD).

Duration of response (DoR) was measured from first objective response until progression or death. Survival was estimated using Kaplan–Meier curves with log‐rank tests for group comparisons, and median follow‐up was calculated with the reverse Kaplan–Meier method.

Resistance to immunotherapy was defined according to the SITC consensus definition: those who progressed within the first 6 months of treatment were considered primary resistant, and the rest of the patients were considered as “previously benefited from immunotherapy” [8].

### 2.3 Statistical Analysis

Two‐sided 95% confidence intervals (95% CI) were calculated for survival analyses, with medians reported as range or interquartile range (IQR). Cox proportional hazard models were used for hazard ratios (HR) and univariable analysis of OS and PFS. For this analysis, variables were selected based on their established prognostic relevance in MM: presence of brain or hepatic metastases, LDH level at baseline, *BRAF* mutation status prior benefit from immunotherapy, previous sequential or combination anti‐PD‐1 exposure, and the reason for treatment discontinuation [5, 9]. Associations with ORR were assessed using chi‐square tests for categorical variables and Mann–Whitney *U* tests for continuous variables.

## 3 Results

### 3.1 Patient Characteristics

From January 1, 2017, to September 9, 2024, a total of 73 patients met the predefined inclusion criteria. Follow‐up continued until September 6, 2025 to ensure at least 12 months of observation for all included patients. The median follow‐up was 27.6 months (range: 12.5–80.0). The median age at treatment initiation was 57.8 years, 58.9% were male, and 94.5% had ECOG performance status of 0 or 1. Most patients had cutaneous melanoma (83.6%), and *BRAF* mutations were present in 86.3% of cases. Nearly half (47.9%) presented with brain metastases at baseline when starting nivolumab/ipilimumab at ≥ third‐line, while 17.8% (13/73) had newly diagnosed brain metastases at that time.

Prior therapies: 93.2% (68/73) had progressed on anti‐PD‐1 treatment, 32.9% (24/73) had received combined or sequential anti‐PD‐1 and anti‐CTLA‐4 therapy, and 98.4% (62/63) of those with *BRAF* mutations had progressed on BRAF/MEK inhibitors.

Treatment was administered as third‐line therapy in 71.2% (52/73) of cases, and 41.1% (30/73) of the total cohort had previously received adjuvant treatment (Table 1). The median interval between the previous treatment and nivolumab/ipilimumab initiation was 1.3 months (IQR 0.95–2.35).

**TABLE 1:** Baseline characteristics of the study population.

Age (median, IQR)	57.8 (48.9–64.4)
Sex	Female: 30 (41.1%) Male 43 (58.9%)
ECOG performance status	0–1: 69 (94.5%) 2–3: 4 (5.5%)
Melanoma (subtype)	Cutaneous: 61 (83.6%) Unknown primary: 10 (13.7%) Mucosal: 2 (2.7%)
*BRAF* status	Wild type: 10 (13.7%) Mutated: 63 (86.3%)
PD‐L1 status	≥ 1%: 24 (32.9%) < 1%: 24 (32.9%) Not tested: 25 (34.2%)
Brain metastases	35 (47.9%)
Liver metastases	22 (30.1%)
LDH (above upper limit of normal)	38 (52.1%)
Previous adjuvant treatment	30 (41.1%)
Previous anti‐PD‐1 (either alone or in combination)	68 (93.2%)
Previous anti‐CTLA‐4 (either alone or in combination)	27 (37.0%)
Previous anti‐PD‐1 and anti‐CTLA‐4	24 (32.9%) Combination: 18 (75.0%) Sequential: 6 (25.0%)
Previous BRAF/MEK inhibitors (% over *BRAF* mutated cases)	62 (98.4%)
Line of therapy	3: 52 (71.2%) 4: 18 (24.7%) 5: 3 (4.1%)
Previous therapies	Neoadjuvant: 0 (0%) Adjuvant: Anti‐PD‐1 monotherapy: 27 (37.0%) Interferon, adjuvant: 2 (2.7%) Dabrafenib/trametinib, adjuvant: 1 (1.4%) Nivolumab/relatlimab: 1 (1.4%)^a^ Metastatic: BRAF/MEK inhibitors: 62 (84.9%) Anti‐PD‐1 monotherapy: 28 (38.4%) Nivolumab/ipilimumab: 18 (24.7%) Ipilimumab: 8 (11.0%) Temozolomide: 7 (9.6%) TIL therapy: 4 (5.5%)^a^ Nivolumab/relatlimab: 1 (1.4%)^a^ Pembrolizumab + vaccine: 1 (1.4%)^a^ TILT‐123: 1 (1.4%)^a^

^a^ Patients treated outside standard of care were included in one of the following protocols: Nivolumab/relatlimab, adjuvant (NCT05002569), nivolumab/relatlimab, metastatic (NCT03715985), pembrolizumab + vaccine (NCT01968109), TUNINTIL (NCT04217473), or a TIL therapy protocol or Early‐access program.

In the group of 24 patients who had received prior anti‐PD‐1 and anti‐CTLA‐4 therapy (either in combination or sequentially), a higher proportion had brain metastases (66.7% vs. 38.8%, *p* = 0.046) and the median age was lower (51.3 vs. 58.2 years, *p* = 0.026). No major differences were observed regarding sex, *BRAF* mutation status, LDH levels, or receipt of adjuvant treatment (Table S1).

### 3.2 Progression‐Free and Overall Survival

**FIGURE 1:**
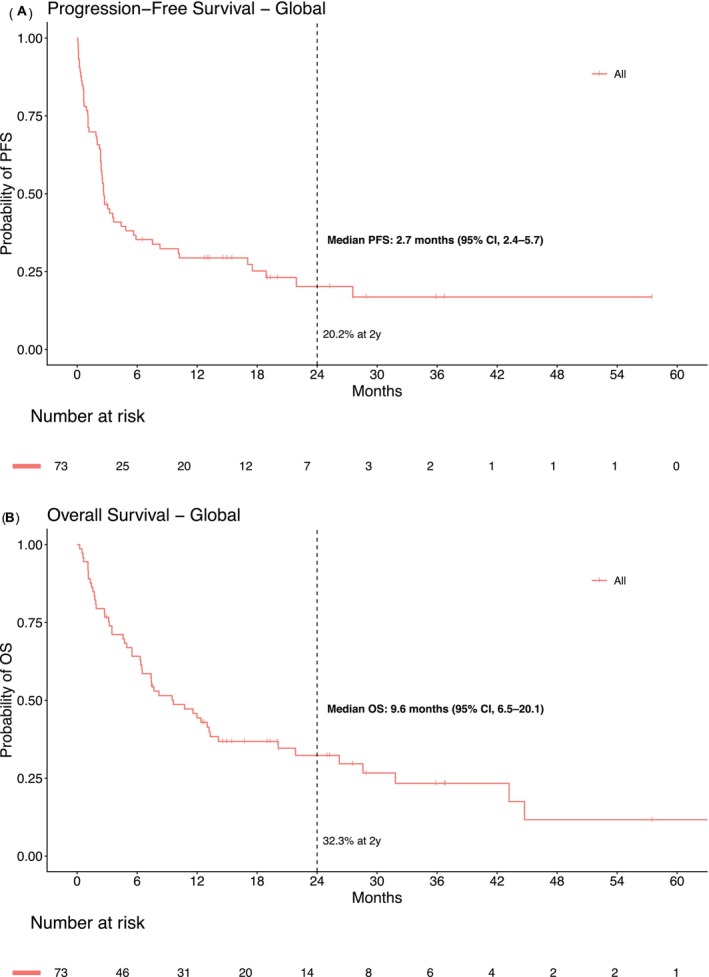
(A) PFS in patients treated with nivolumab/ipilimumab in third line setting and beyond. (B) OS in patients treated with nivolumab/ipilimumab in third line setting and beyond.

The median PFS of the whole cohort was 2.7 months (95% CI, 2.4–5.7), with a 2‐year PFS rate of 20.2% (Figure 1). PFS was similar according to the presence of brain metastases (*p* = 0.357) (Figure S1), liver metastases (*p* = 0.367), *BRAF* mutation status (*p* = 0.521), or elevated LDH (*p* = 0.852).

The median OS was 9.6 months (95% CI, 6.5–20.1), with a 2‐year OS rate of 32.3% (Figure 1B). As with PFS, we did not observe a major difference in OS according to baseline brain metastases (*p* = 0.271), liver metastases (*p* = 0.117), *BRAF* mutation status (*p* = 0.295), or elevated LDH (*p* = 0.693).

No major differences in PFS (*p* = 0.790) or OS (*p* = 0.723) were observed according to prior exposure to anti‐CTLA‐4 and anti‐PD‐1 therapy, sequential or in combination (Figure S2). A trend toward improved survival was observed in patients with previous benefit to immunotherapy (PFS, *p* = 0.082; OS, *p* = 0.161). By contrast, no differences were identified among patients who had discontinued prior immunotherapy due to progression, toxicity, or treatment completion (PFS, *p* = 0.766; OS, *p* = 0.778) (Figure S3).

### 3.3 Tumor Responses

**FIGURE 2:**
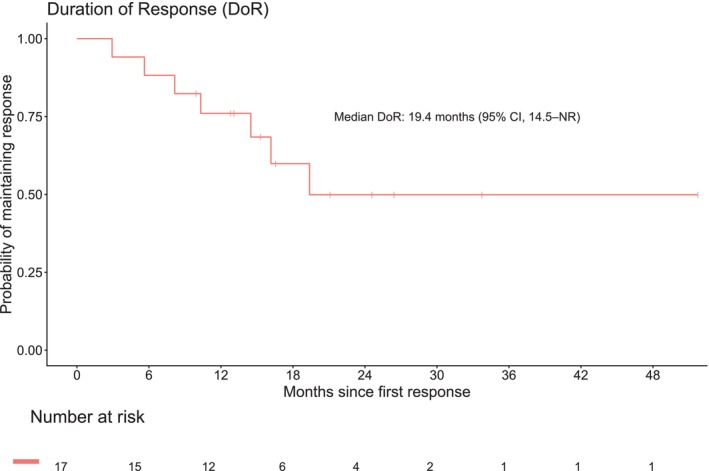
DoR in responders treated with nivolumab/ipilimumab in third line setting and beyond.

In the overall cohort, the ORR was 23.3% (17/73), with a DCR of 32.9% (24/73). CR was observed in 6.8% (5/73) of patients, and the median DoR was 19.4 months (95% CI, 14.5‐NR) (Figure 2). Median OS in the group of responders was 44.7 months (95% CI, 28.6‐NR).

**FIGURE 3:**
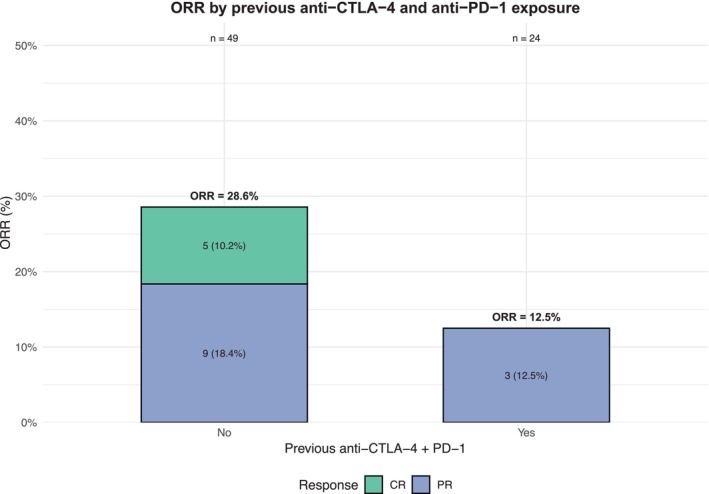
ORR according to previous progression to anti‐PD‐1 and anti‐CTLA‐4 treatment.

When analyzed by prior exposure to anti‐PD‐1 and anti‐CTLA‐4 therapy (either combined or sequential), the ORR was 12.5% (3/24), compared with 28.6% (14/49) in patients without previous exposure to both anti‐PD‐1 and anti‐CTLA‐4 (*p* = 0.22) (Figure 3). Within this subgroup, outcomes were similar between sequential and combination strategies, with ORRs of 16.7% (1/6) and 11.1% (2/18) (*p* = 1), respectively, and no CRs were observed in either of the groups.

No major associations with ORR were observed for the presence of brain metastases (*p* = 0.583), PD‐L1 status (*p* = 0.503), liver metastases (*p* = 1.000), elevated LDH (*p* = 0.517), or sex (*p* = 0.272). Similarly, no major association was found between patients' responses to prior therapy and to reinduction. Figure S4 shows that BOR at re‐treatment did not correlate with initial response to nivolumab/ipilimumab. Outcomes after reinduction were heterogeneous across all prior BOR categories, with no evidence of concordance (*p* = 0.904).

Patients with previous benefit from immunotherapy had a numerically higher ORR compared to those with primary resistance (26.1% (6/23) vs. 15% (6/40), *p* = 0.328).

### 3.4 Toxicity

12/73 patients (16.4%) discontinued treatment due to toxicity‐related reasons.

## 4 Discussion

This nationwide retrospective cohort study demonstrates that nivolumab/ipilimumab can provide a durable benefit in a subset of patients with heavily pre‐treated MM, even with brain metastases. When responses occurred, they often translated into meaningful clinical benefit, with a median duration of response exceeding 1.5 years. However, OS outcomes in the whole cohort were poor, with short median PFS and OS and ORR below 25%. These results align with the ORR of previously reported cohorts in anti‐PD‐1‐resistant disease, with ORR of 12%–31% [2, 3]. However, those cohorts included fewer heavily pre‐treated patients, with only 17%–23% receiving therapy in the third line or beyond.

As expected, outcomes were worse in patients previously treated with both anti‐PD‐1 and anti‐CTLA‐4, particularly in those who had already progressed on combined nivolumab/ipilimumab, where the ORR was ~10% and no CRs were observed. This ORR was also lower than previously reported in patients with rechallenge (23%–26%), likely reflecting its use in later lines in our study, while the previously published cohorts included fewer patients with *BRAF*‐mutant melanoma and provided limited information on prior therapies [10, 11]. Previous treatment with BRAF/MEK inhibitors could have also determined a lower response rate [12, 13], but this fact cannot be assessed in this study given the predominance of *BRAF* mutated cases given the nature of its design.

Notably, this difference may partly reflect the higher incidence of brain metastases in the group that had previously received the combination. Consistent with our findings, those studies also demonstrated that neither prior benefit nor the reason for discontinuation predicted later response upon re‐induction. Notably, some patients without prior benefit may achieve responses or disease stabilization when rechallenged, indicating that the absence of initial benefit does not necessarily exclude activity at a later line.

In the absence of validated predictive biomarkers, many patients may be exposed to a toxic regimen without achieving meaningful benefit. Translational data suggest that *NRAS* mutations and the expansion of CD4+ T‐cell subsets may correlate with benefit from ipilimumab‐based therapy [2, 14], but whether this applies to late lines or retreatment settings remains unknown.

Our findings need to be interpreted in the context of an evolving third‐line landscape. Nivolumab/relatlimab is now a first‐line option across several countries, even though in some regions only approved for patients with PD‐L1 negative disease [15], yet fewer than 5% of patients in our cohort had been treated with this regimen, limiting generalization to anti‐LAG‐3‐based therapies. TIL therapy is also available for selected patients before third line, although only 5.5% of our cohort had previously undergone this approach. In addition, novel intratumoral agents and antiangiogenic agents have recently shown promising results in phase II single‐arm trials in the second‐line setting, and may potentially emerge as therapeutic options in the future, further complicating the therapeutic landscape [16, 17, 18]. It remains unknown whether these novel agents modulate the immune microenvironment to increase or diminish sensitivity to nivolumab/ipilimumab.

Nevertheless, our study has some inherent limitations. The retrospective design may introduce bias, and so the design is reflected in the high prevalence of *BRAF* mutated cases. Furthermore, the cohort remains small for detecting meaningful differences between subgroups or identifying reliable predictors of benefit. As the sample size was fixed, no formal power calculation was feasible, and *p* values should be interpreted descriptively. These limitations should be considered when interpreting our findings.

## 5 Conclusion

In conclusion, in a real‐world setting, nivolumab/ipilimumab beyond the second line achieved durable benefit in a subset of patients. Yet, overall outcomes were poor; prior exposure to both anti‐PD‐1 and anti‐CTLA‐4 markedly reduced response likelihood, and prior benefit did not predict reinduction outcomes. These findings underscore the urgent need for novel therapeutic approaches and refined patient selection in late‐line melanoma.

## Author Contributions

**Yago Garitaonaindia:** writing – original draft, project administration, methodology, investigation, software, formal analysis, data curation, visualization. **Søren Kjær Petersen:** writing – review and editing, project administration, resources. **Louise M. Guldbrandt:** writing – review and editing, project administration, resources. **Troels H. Borch:** writing – review and editing, project administration, resources. **Christina H. Ruhlmann:** writing – review and editing, project administration, resources. **Rasmus Blechingberg Friis:** writing – review and editing, project administration, resources. **Adam A. Luczak:** writing – review and editing, project administration, resources. **Lars Bastholt:** writing – review and editing, project administration, resources. **Henrik Schmidt:** writing – review and editing, project administration, resources. **Inge Marie Svane:** writing – review and editing, project administration, resources, funding acquisition, conceptualization. **Eva Ellebaek:** writing – review and editing, writing – original draft, supervision, visualization, project administration, methodology, investigation, formal analysis, data curation, conceptualization. **Marco Donia:** writing – review and editing, writing – original draft, investigation, conceptualization, methodology, visualization, formal analysis, data curation, supervision, project administration.

## Funding

This work was supported by the Danish Metastatic Melanoma Database (DAMMED), which is supported financially by MSD, BMS, Novartis, and Pierre Fabre without influencing data collection or results.

## Ethics Statement

This study was conducted in accordance with the ethical standards of the Helsinki Declaration and applicable national regulations. The project was approved by the Capital Region Data Protection Officer (reference number: P‐2023‐14211), and all patients registered in DAMMED have signed informed consent.

## Conflicts of Interest

The authors declare the following financial interests and personal relationships, which may be considered as potential competing interests. Yago Garitaonaindia received honoraria for lectures and travel grants from Regeneron, Roche, Pierre‐Fabre, and Bristol‐Myers Squibb. Søren Kjaer Petersen reports travel and conference support by MSD. Rasmus Blechingberg Friis received honoraria for travel expenses from MSD and AstraZeneca and for advisor and presenter roles from BMS. Troels H. Borch received a speaker's fee from Bristol Myers Squibb and MSD Denmark. Christina H. Ruhlmann has received institutional grants from Novo Nordisk Foundation and Helsinn Healthcare SA and personal honoraria from Bristol‐Myers Squibb, AstraZeneca, GSK, and Novartis. Louise M. Guldbrandt received honoraria for lectures from Bristol‐Myers Squibb and from AstraZeneca. Inge Marie Svane reports personal payments (honoraria) for lectures, presentations, speakers' bureaus, manuscript writing, advisory boards, or educational events received from MSD, Takeda, Sanofi Aventis, Janssen Cilag, and BMS. Institutional grants and contracts were received from Evaxion Biotech, Adaptimmune, IO Biotech, Asgard Biotech, TILT Biotherapeutics, and Enara Bio. Consulting fees were received from TILT Biotherapeutics, IO Biotech, Novartis, and Genmab. She holds stocks/shares in IO Biotech and received meeting support from MSD. Additionally, she received clinical trial drugs (Relatlimab) from BMS and participates in DSMB for academic trials only. Marco Donia has received advisory fees from Achilles Therapeutics and NeoGap Therapeutics and consultancy fees via membership of Guidepoint LLC and Alphasights expert network. Eva Ellebaek received a speaker's fee from Novartis, Pierre Fabre, Bristol Myers Squibb, and MSD Denmark and travel and conference support from Pierre Fabre and MSD Denmark. The other authors declare no conflicts of interest.

## Supporting information


**Table S1:** Baseline characteristics according to prior anti‐CTLA‐4/PD‐1 exposure.
**Figure S1:** PFS stratified by the presence of brain metastases.
**Figure S2:** PFS and OS according to previous exposure to anti CTLA‐4 and anti‐PD‐1.
**Figure S3:** Univariable Cox regression for PFS and OS.
**Figure S4:** Concordance of response between first and the later course of nivolumab/ipilimumab. Only patients treated with the combination were included (not in sequence).

## Data Availability

The data that support the findings of this study are derived from the Danish Metastatic Melanoma Database (DAMMED). Due to Danish data protection regulations and confidentiality constraints, the raw individual‐level data are not publicly available. Aggregated de‐identified data may be provided by the corresponding author upon reasonable request and subject to approval by the DAMMED steering committee and applicable legal/ethical regulations. Further information is available from the corresponding author upon request.
