# Supplementary material for: Real‐World Outcomes of Nivolumab and Ipilimumab in Metastatic Melanoma as Third Line and Beyond
Source: Int J Cancer. 2026 Apr 28;159(7):1780–7. doi: 10.1002/ijc.70520 (PMC13432422; doi:10.1002/ijc.70520)

SUPPLEMENTARY MATERIAL:

TITLE: Real-world Outcomes of Nivolumab and Ipilimumab in Metastatic Melanoma as Third Line and Beyond

Authors: Yago Garitaonaindia, Søren Kjær, Louise M. Guldbrandt, Troels H. Borch, Christina H. Ruhlmann, Rasmus Blechingberg Friis, Adam A. Luczak, Lars Bastholt, Henrik Schmidt, Inge Marie Svane, Eva Ellebaek, Marco Donia

Table of contents:

Table S1. Baseline characteristics according to prior anti-CTLA-4/PD-1 exposure

Figure S1. PFS stratified by the presence of brain metastases

Figure S2. PFS and OS according to previous exposure to anti CTLA-4 and anti-PD-1

Figure S3. Univariable Cox Regression for PFS and OS

Figure S4. Concordance of response between first and the later course of Nivolumab/ipilimumab. Only patients treated with the combination were included (not in sequence)

Table S1. Baseline characteristics according to prior anti-CTLA-4/PD-1 exposure

	**No** *N=49*	**Yes** *N=24*	**P value**
Sex:			0.407
Female	18 (36.7%)	12 (50.0%)	
Male	31 (63.3%)	12 (50.0%)	
BRAF status:			0.687
Wild type	5 (10.2%)	5 (20.8%)	
Mutated	44 (89.8%)	19 (79.2%)	
PD-L1 status	≥1%: 18 (36.7%)<br><1%: 16 (32.7%)<br>Not tested: 15 (30.6%)	≥1%: 6 (25.0%)<br><1%: 8 (33.3%)<br>Not tested: 10 (41.7%)	0.517
Brain metastases:			0.046*
No	30 (61.2%)	8 (33.3%)	
Yes	19 (38.8%)	16 (66.7%)	
Liver metastases:			0.138
No	31 (63.3%)	20 (83.3%)	
Yes	18 (36.7%)	4 (16.7%)	
LDH (Upper Limit of Normal):			0.923
No	23 (47.9%)	10 (43.5%)	
Yes	25 (52.1%)	13 (56.5%)	
Age	58.2 (11.9)	51.3 (12.1)	0.026*
Adjuvant treatment:			0.220
No	28 (57.1%)	18 (75.0%)	
Yes	21 (42.9%)	6 (25.0%)	

Figure S1. PFS stratified by the presence of brain metastases

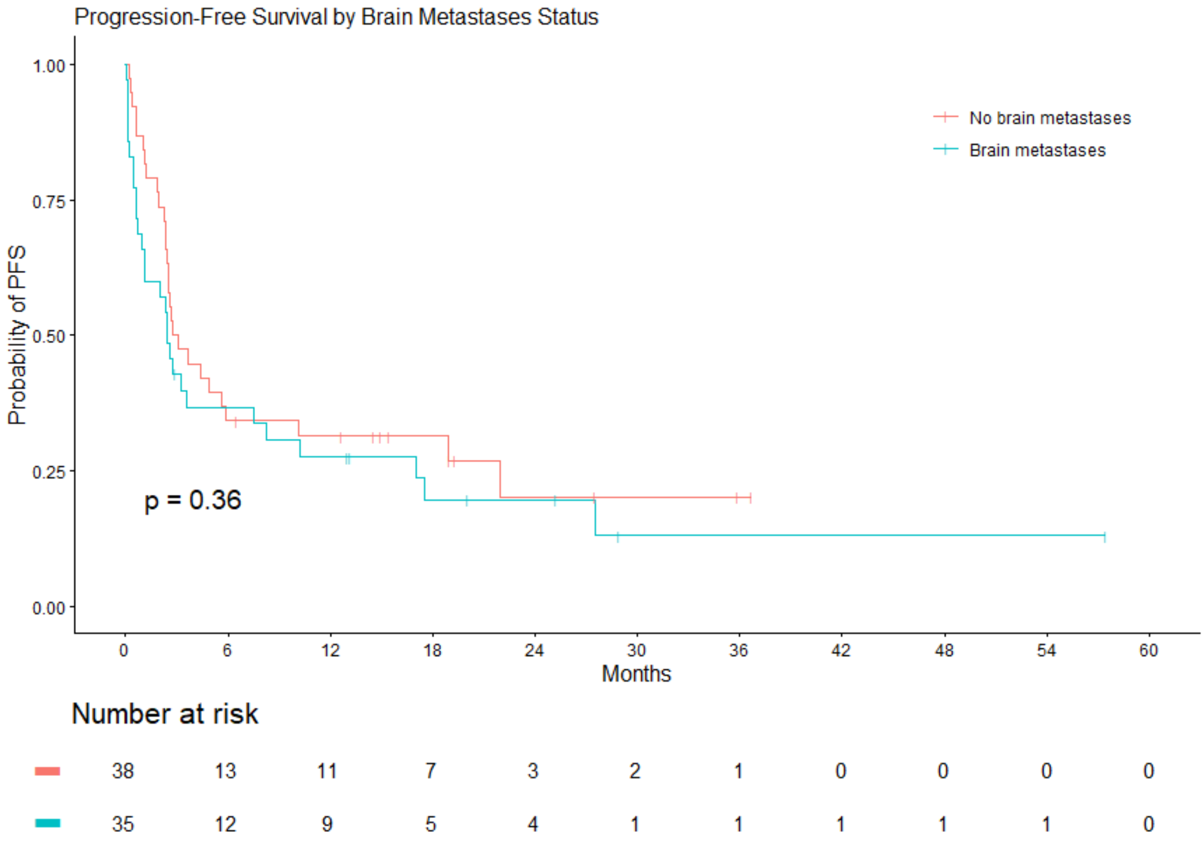
Progression-Free Survival by Brain Metastases Status
1.00
0.75
0.50
0.25
0.00
Probability of PFS
No brain metastases
Brain metastases
p = 0.36
0
6
12
18
24
30
36
42
48
54
60
Months
Number at risk
38 13 11 7 3 2 1 0 0 0 0
35 12 9 5 4 1 1 1 1 1 0

Figure S2. PFS and OS according to previous exposure to anti CTLA-4 and anti-PD-1

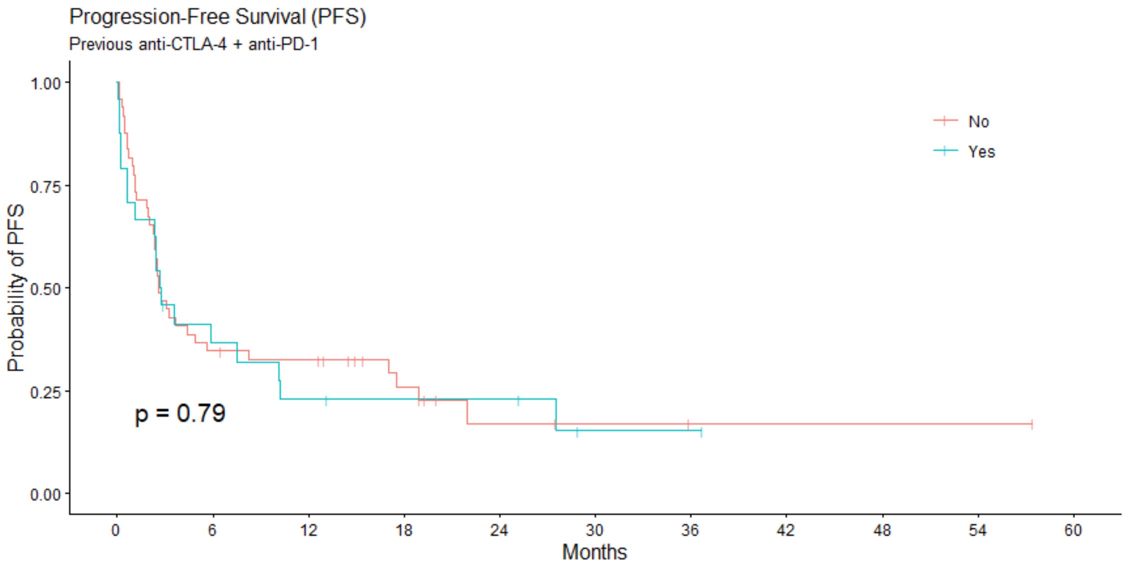

Number at risk

	0	6	12	18	24	30	36	42	48	54	60
No	49	17	15	8	3	2	1	1	1	1	0
Yes	24	8	5	4	4	1	1	0	0	0	0

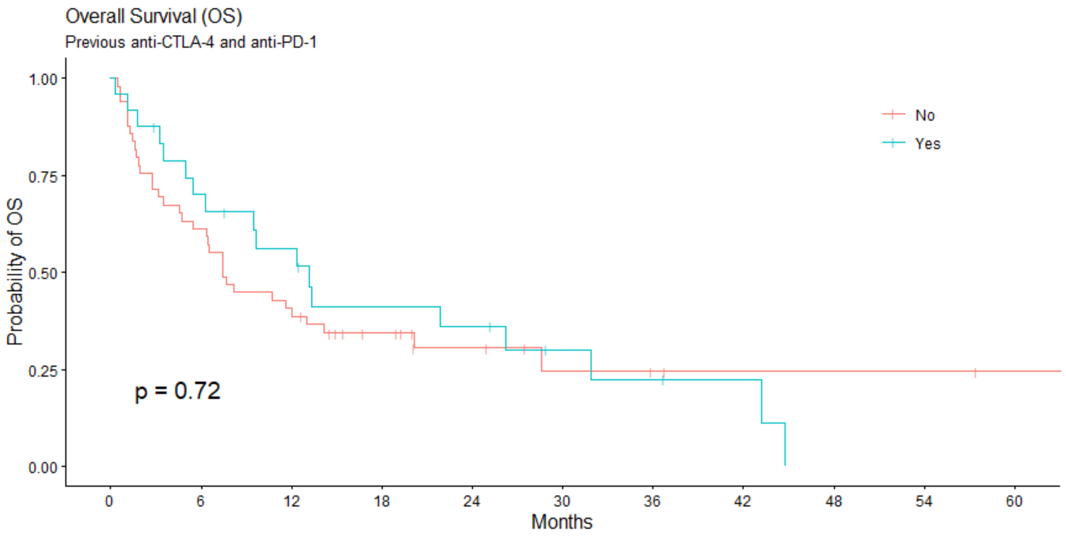

Number at risk

	0	6	12	18	24	30	36	42	48	54	60
No	49	30	19	12	7	4	3	2	2	2	1
Yes	24	16	12	8	7	4	3	2	0	0	0

Figure S3. Univariable Cox Regression for PFS and OS

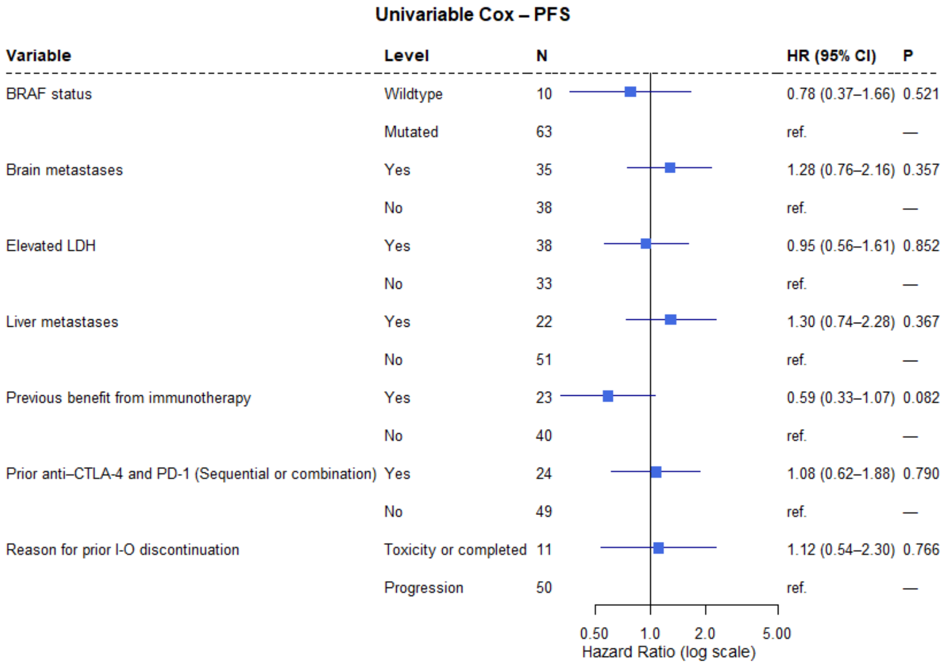

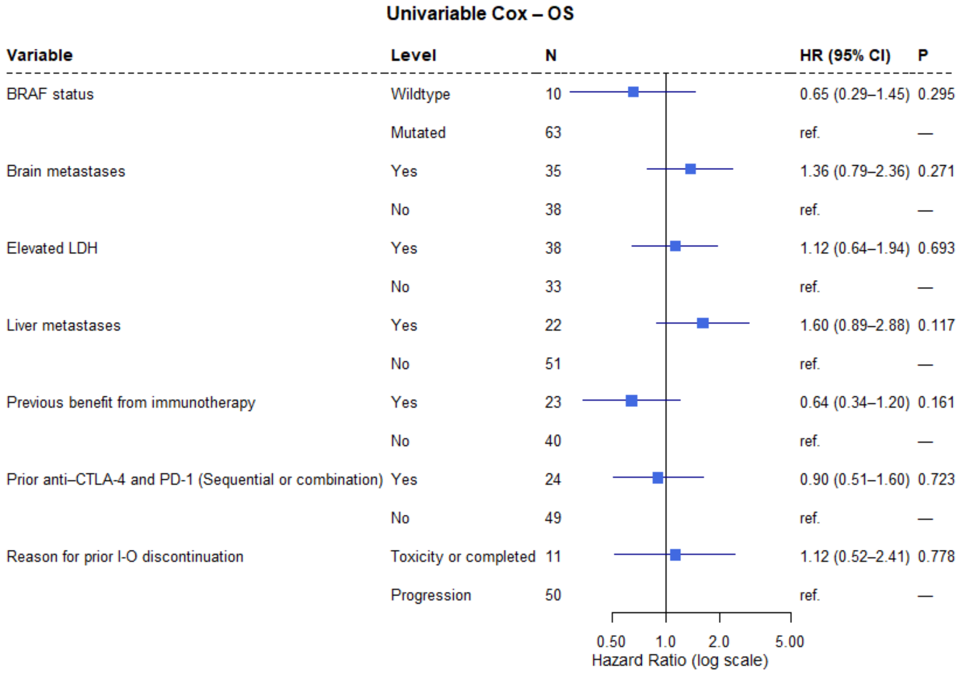

Figure S4. Concordance of response between first and the later course of Nivolumab/ipilimumab. Only patients treated with the combination were included (not in sequence)

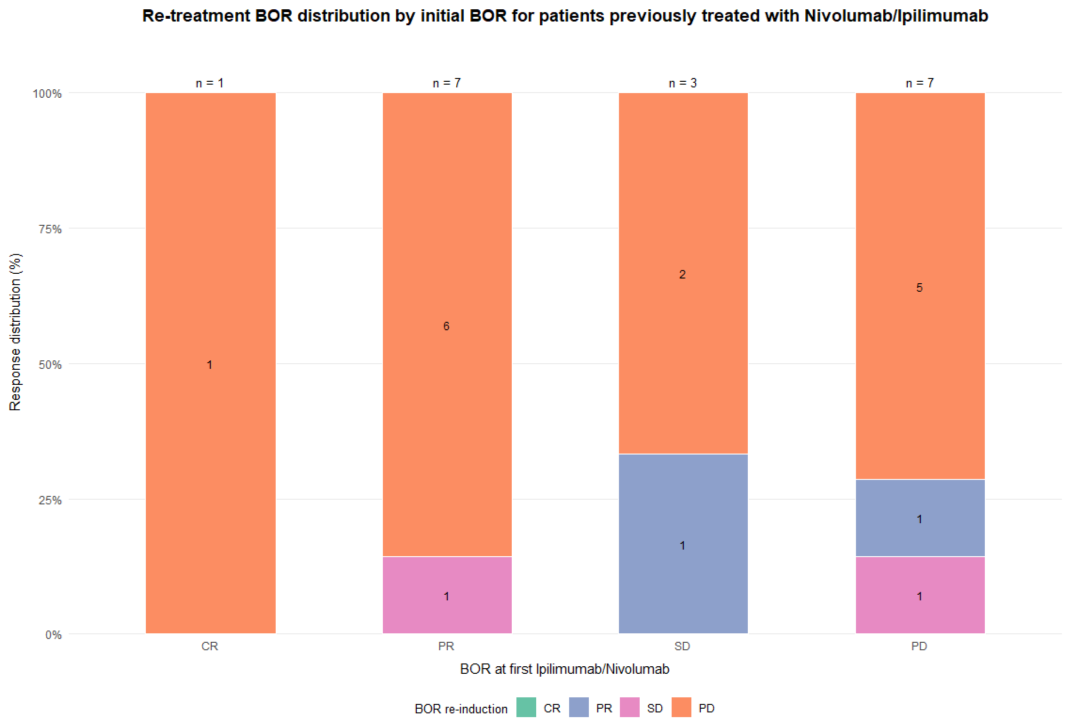

Supplement: Supplementary file 1 — Table S1: Baseline characteristics according to prior anti‐CTLA‐4/PD‐1 exposure. Figure S1: PFS stratified by the presence of brain metastases. Figure S2: PFS and OS according to previous exposure to anti CTLA‐4 and anti‐PD‐1. Figure S3: Univariable Cox regression for PFS and OS. Figure S4: Concordance of response between first and the later course of nivolumab/ipilimumab. Only patients treated with the combination were included (not in sequence). [file IJC-159-1780-s001.pdf]
